# Cellular mechanisms underlying the effects of an early experience on cognitive abilities and affective states

**DOI:** 10.1186/1744-859X-4-8

**Published:** 2005-04-06

**Authors:** Efstathios Garoflos, Theofanis Panagiotaropoulos, Stavroula Pondiki, Antonios Stamatakis, Eleni Philippidis, Fotini Stylianopoulou

**Affiliations:** 1 Lab. Biology-Biochemistry, Dept. Basic Sciences, Faculty of Nursing, University of Athens, Papadiamantopoulou 123, 115 27 Athens, Greece

## Abstract

In the present study we investigated the effects of neonatal handling, an animal model of early experience, on spatial learning and memory, on hippocampal glucocorticoid (GR), mineralocorticoid (MR) and type 1A serotonin (5-HT1A) receptors, as well as brain derived neurotrophic factor (BDNF), and on circulating leptin levels, of male rats.

**Method:**

Spatial learning and memory following an acute restraint stress (30 min) were assessed in the Morris water maze. Hippocampal GR, MR and BDNF levels were determined immunocytochemically. 5-HT1A receptors were quantified by in vitro binding autoradiography. Circulating leptin levels, following a chronic forced swimming stress, were measured by radioimmunoassay (RIA). Data were statistically analyzed by analysis of variance (ANOVA).

**Results:**

Neonatal handling increased the ability of male rats for spatial learning and memory. It also resulted in increased GR/MR ratio, BDNF and 5-HT1A receptor levels in the hippocampus. Furthermore, leptin levels, body weight and food consumption during chronic forced swimming stress were reduced as a result of handling.

**Conclusion:**

Neonatal handling is shown to have a beneficial effect in the males, improving their cognitive abilities. This effect on behavior could be mediated by the handling-induced increase in hippocampal GR/MR ratio and BDNF levels. The handling-induced changes in BDNF and 5-HT1A receptors could underlie the previously documented effect of handling in preventing "depression". Furthermore, handling is shown to prevent other maladaptive states such as stress-induced hyperphagia, obesity and resistance to leptin.

## Background

It is generally accepted that early experiences have profound influences on brain development and thus on adult brain function and behavior. However the neurobiological mechanisms involved still remain elusive. An animal model employed in experiments aiming to elucidate such mechanisms is "neonatal handling" [1]. This manipulation alters hypothalamic-pituitary-adrenal (HPA) axis function and the ability of the organism to respond to stressful stimuli [1]. Thus, as adults, neonatally handled rats are less emotionally reactive, synthesize and secrete less corticotropin-releasing factor, adrenocorticotropin hormone (ACTH) and corticosterone following a variety of stressors [2], and their stress-induced secretion is more short-lived [3]. These differences in HPA axis reactivity have been attributed to an enhanced sensitivity of the negative-feedback loop [2], due to a handling-induced increase in the number of type II glucocorticoid receptors (GR) in the hippocampus [2].

In addition to GR, glucocorticoids also bind to type I (MR) receptors, and the hippocampus is rich in both these types of receptors [4]. GR and MR receptors are the molecules mediating the negative feedback control exerted by glucocorticoids on HPA axis function [5]. Furthermore GRs and MRs influence spatial learning, a process controlled by the hippocampus [6]. MRs have a role in behavioral reactivity during novel situations [7], whereas GRs are involved in consolidation of learned information. In addition to GRs and MRs, glucocorticoid levels also play a determinant role in the ability for learning and memory. The effect of corticosteroid levels on cognition exhibits a U-shaped dose-response dependency [8]. Interestingly, as mentioned above, handled animals have lower corticosterone levels following stress [9], which could alter their ability for learning and memory.

Another molecule that has been shown to play a key-role in the cellular processes underlying learning and memory is Brain Derived Neurotrophic Factor (BDNF), a member of the neurotrophin family [10]. BDNF mRNA is increased during LTP, indicating that BDNF is involved in plastic changes of neuronal function [11]. Memory acquisition is also associated with increased BDNF mRNA and activation of its receptor TrkB [12,13]. On the other hand, LTP is markedly impaired in BDNF mutant mice and the deficit is restored by the re-expression of BDNF [14,15]. Moreover, BDNF mutant mice show learning deficits [16]. Similarly, the pharmacologic deprivation of BDNF or its receptor TrkB, results in severe impairment of learning and memory in mice, rats and chicks [15]. BDNF mutant mice develop enhanced aggressiveness, and hyperphagia, accompanied with weight gain in early adulthood, findings reminiscent of dysfunction of the serotoninergic system [17]. Indeed BDNF is known to have trophic effects on serotoninergic neurons [18]. It is well known that depression is associated with hypofunctioning of the serotoninergic system. Recently BDNF has emerged as a major factor in the pathophysiology of depression: BDNF mRNA is increased in the rat brain following chronic anti-depressant or electro-convulsive shock treatment [19,20]. Administration of BDNF in the hippocampus has been shown to have an anti-depressant effect in the forced swimming and learned helplessness paradigm [21]. Furthermore, in patients with major depression, serum BDNF levels were decreased, while hippocampal BDNF immunoreactivity was increased in post-mortem tissues from subjects treated with anti-depressants [22]. Previous results from our laboratory have shown that handled males exhibit decreased expression of "depressive" behavior [26].

Recent evidence indicates that among the serotonin receptors, the type 1A are involved in the etiopathogenesis of certain types of depression [23,24] and is the one through which the therapeutic effects of the Selective Serotonin Re-uptake Inhibitors (SSRIs), a major class of antidepressants, are mediated [25]. Results from our laboratory have shown that handled male rats show increased 5-HT1A receptor sensitivity as assessed by the hypothermic response to 8-OH-DPAT compared to the non-handled [26].

Depression and the response to chronic stress are often associated with disorders in food-intake behavior, which is influenced by serotonin and, as mentioned above, by BDNF. A key hormone regulating food-intake behavior is leptin, the product of the *ob *gene [27]. Leptin, whose levels reflect the organism's current energy balance, is secreted from adipose tissue proportionally to body fat mass and acts on the CNS to limit food intake, and thus promote body weight loss [28]. Recent evidence indicates that glucocorticoids induce leptin synthesis and secretion and that, conversely, leptin participates in the regulation of HPA axis function [29].

Thus, we investigated the effects of "neonatal handling" on factors influencing cognitive abilities and affective states of the adult rat. Specifically, we determined the "neonatal handling" effects on A. the ability for spatial learning and memory -in the Morris water maze- when a short-term restrain stress has preceded the learning process, B. GR and MR levels in the hippocampus after the completion of the Morris water maze test, C. BDNF levels in the hippocampus, D. hippocampal 5-HT1A receptor density and E. plasma leptin levels, food intake and body weight change during long term forced swimming stress.

## Methods

### Animals

Male Wistar rats reared in our laboratory were kept under standard conditions (24°C; 12:12 h light/dark cycle; food and water *ad libitum*). All animal experimentations were carried out in agreement with ethical recommendation of the European Communities Council Directive of 24 November 1986 (86/609/EEC). In total, 43 handled and 45 non-handled (control) animals were used in this study.

### Neonatal handling

Pups were removed from their mothers and placed for 15 min in a plastic container lined with paper towel, daily from postnatal day 1 until weaning (postnatal day 22). The non-handled animals were left completely undisturbed until weaning.

### Restraint stress

Adult, handled and non-handled males were placed in a cylinder 15 cm in length and 5 cm in diameter for 30 min.

### Spatial learning and memory test

The Morris water maze (MWM) apparatus was a circular galvanized tank (1.38 m in diameter, 0.5 m in height), filled to a depth of 28 cm with water (24°C), made opaque with milk. The training session took place 90 min after the completion of the restraint stress. For this session a 2 cm submerged platform (13 × 13 cm) was placed in a fixed position. The single training session consisted of 8 trials with 4 different starting positions. After finding the platform, the animals were allowed to remain on it for 20 sec and were then placed in a holding cage for 30 sec until the beginning of the next trial. The testing trial was performed 24 hours later. It consisted of a 60 sec free swim period without a platform and was recorded on videotape. The rat was placed in the tank at a position directly opposite to the imaginary platform quadrant. Animals were sacrificed upon termination of the testing session and their brains were used for GR and MR immunocytochemistry.

### Immunocytochemistry

For the GR and MR immunocytochemistry the same animals were used, whereas for the BDNF immunocytochemistry a different set of animals was employed. All animals were deeply anesthetized with ether and perfused transcardially with 4% paraformaldehyde in 0.1 M phosphate buffer (PB). Immunocytochemistry was performed as previously reported [30] on paraffin, sagittal brain sections (6 μm). The primary antibodies used were an anti-BDNF rabbit polyclonal antibody (Santa Cruz) or an anti-MR goat polyclonal antibody (Santa-Cruz) or an anti-GR moloclonal antibody (kindly provided by Dr. Alexis, NHRF). The secondary antibodies were biotinylated goat anti-rabbit or rabbit anti-goat or rabbit anti-mouse antibody respectively (DAKO). Staining of the immunopositive cells was performed using the DAKO ABC reagent followed by the 3,3'-diaminobenzidine (DAB) reaction. The number of immunopositive cells was evaluated using Image-Pro Plus program (Media Cybernetics, USA), in 3–5 sections from each brain, and an average value was calculated for each of the areas studied per animal.

### In vitro binding

Animals used for 5-HT1A receptor autoradiogarphy were killed by decapitation under ether anesthesia. Their brains were frozen at -40°C in dry-ice cooled isopentane and subsequently cut coronally (10 μm) in a cryostat (-17°C). The sections were processed using standard autoradiographic procedures [31,32]. Briefly, the localization of 5-HT1A receptors was performed using 4 nM ^3^H-8-OH-DPAT (129Ci/mmol, NEN) and non-specific binding was determined in the presence of 10 μM serotonin. Bound ^3^H-8-OH-DPAT was visualized by exposing the labeled sections to tritium-sensitive film (Biomax, KODAK) (4oC, 1 month) along with ^3^H-standards (^3^H-microscales, ARC). Quantitative image analysis of the autoradiograms was performed using SCION-Image for Windows. Specific binding, >95% of the total binding, was expressed as fmol/mgr tissue.

### Long term forced swimming

On each of 15 consecutive days adult handled and non-handled male animals were placed for 5 min in a glass cylinder 33 cm in height and 20 cm in diameter containing tap water at 24°C.

### Body weight measurement

During the period of the long term forced swimming handled and non-handled animals were weighed daily prior to the exposure to the stressful stimulus. Moreover, the amount of food consumed daily was determined for each one of these animals.

### Determination of plasma leptin levels

Immediately after the last exposure to long term forced swimming (day 15) blood samples from all animals were collected by cardiac puncture under ether anesthesia, using heparinized syringes, and centrifuged to obtain plasma. Leptin concentrations were determined by RIA (Linco's™ rat leptin [^125^I] assay system).

### Statistical Analysis

Data were analyzed by a one-way analysis of variance (ANOVA) with handling as the independent factor. Data on learning, body weight and food intake were analyzed by a one-way ANOVA with repeated measures (handling served as the independent factor and days of training served as the repeated factor). All tests were performed with the software SPSS for Windows (10.0.1, SPSS Inc.). Differences were considered as significant if p < 0.05.

## Results

**Figure 1:**
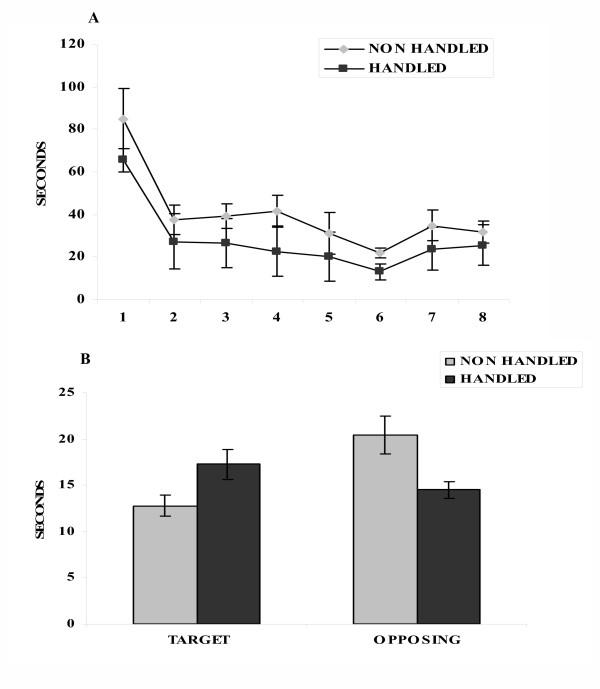
**Effects of handling on spatial learning and memory in the Morris water maze following an acute restraint stress. **A. Mean escape latencies-Learning: handled animals took less time to find the submerged platform during the 8 learning trials compared to the non handled (p = 0.05, one way ANOVA with repeated measures). Values represent mean escape latencies ± S.E.M.B. Memory: handled animals spent more time in the target and less in the opposing quadrant compared to the non handled (p = 0.024, one way ANOVA with repeated measures). Values represent the mean time spent in each quadrant ± S.E.M.

**Figure 2:**
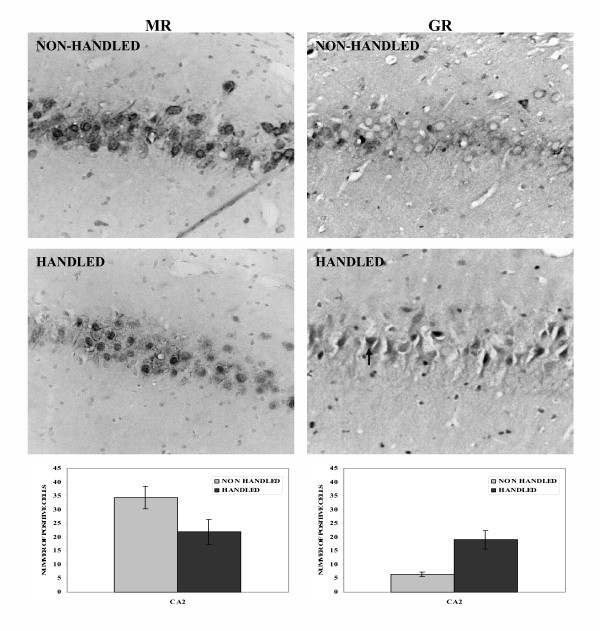
**Effects of handling on MR and GR immunoreactivity in the CA2 region of the hippocampus. **Handling decreased the number of MR positive cells (p = 0.042, one way ANOVA) but increased the number of GR positive cells (p = 0.002, one way ANOVA) in the CA2 region of the hippocampus. The arrow points to a GR positive cell. Values represent means ± S.E.M.

Following exposure to a short term restraint stress handled animals displayed a greater ability for spatial learning in the Morris water maze, as shown by the lower mean escape latencies (time to find the submerged platform) of the handled animals during the acquisition of the task (F_1,15 _= 4.565, p = 0.05) (Fig. 1A). Furthermore, handled animals spent more time in the target, and less in the opposing quadrant during the probe trial (F_1,15 _= 6.320, p = 0.024) (Fig. 1B), indicating superior mnemonic function (better consolidation of information). The effects of "neonatal handling" on cognition were accompanied by changes in GR and MR hippocampal levels: Higher GR and lower MR levels were found in the CA2 region of the hippocampus of handled, compared to the non-handled animals, following their exposure to the Morris water maze (F_1,13 _= 14.632, p = 0.002 and F_1,13 _= 5.268, p = 0.042, respectively) (Fig. 2).

**Figure 3:**
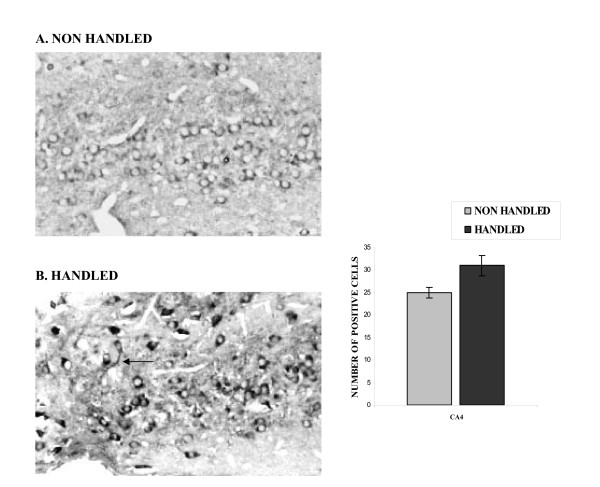
**Effect of handling on BDNF immunoreactivity in the CA4 region of the hippocampus. **Handling resulted in increased number of BDNF positive cells in the CA4 (p < 0.001, one way ANOVA) region of the hippocampus. The arrow points to a neuronal process. Values represent the mean number of BDNF positive cells ± S.E.M

"Neonatal handling" resulted in increased number of BDNF immunopositive cells, in the CA4 region of the hippocampus (F_1,13 _= 35.388, p < 0.001) (Fig. 3). BDNF immunoreactivity was clearly localized in the cytoplasm. The BDNF positive cells were large, with typical neuronal morphology, including processes (see arrow).

**Figure 4:**
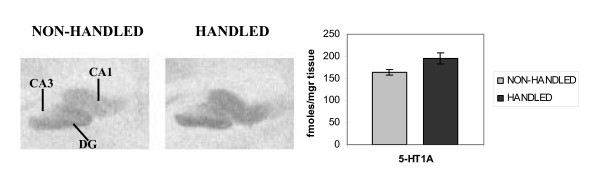
**Effects of handling on the density of 5-HT1A receptors in the hippocampus. **Neonatal handling increased the number of ^3^H-8-OH-DPAT binding sites in the hippocampus (p = 0.027, one way ANOVA), indicating an increased density of 5-HT1A receptors in this area. Values represent the mean ± S.E.M. of 5-HT1A receptor density in fmoles/mgr tissue.

"Neonatal handling" increased the density of 5-HT1A receptors in the hippocampus (areas CA1, CA2, CA4 and DG) as revealed by ^3^H-8-OH-DPAT binding (F_1,13 _= 9.170, p = 0.027). Notably, the CA3 region was devoid of any detectable labeling (Fig. 4).

**Figure 5:**
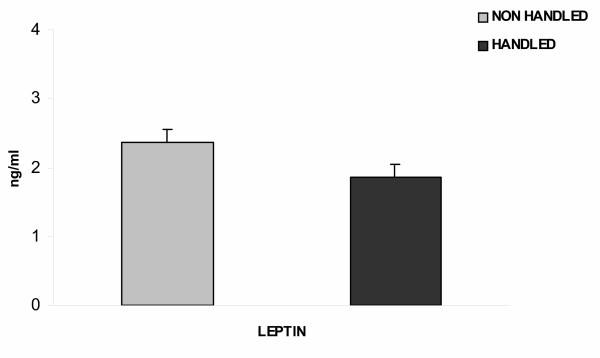
**Effect of handling on leptin secretion following long term forced swimming stress. **Handled animals had lower plasma leptin levels after long term forced swimming, (p = 0.047, one way ANOVA). Values represent mean leptinlevels ± S.E.M

**Figure 6:**
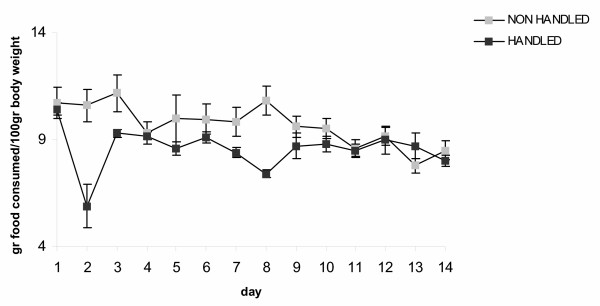
**Effect of handling on food consumption during long term forced swimming stress. **Handled animals consumed less food during long term forcedswimming (p = 0.05, one way ANOVA). Values represent the mean of food consumed in gr/100 gr body weight ± S.E.M

**Figure 7:**
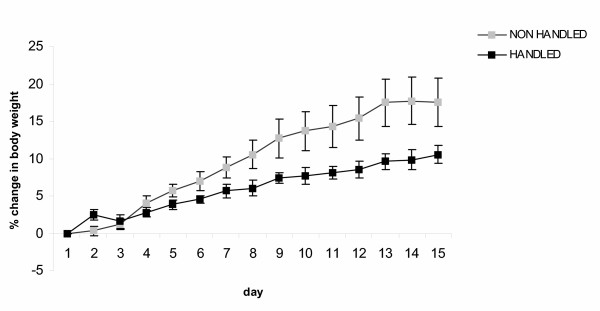
**Effect of handling in body weight change during long term forced swimming stress. **Handled animal gained less weight during long-term forced swimming stress compared to the non-handled (p = 0.017, one way ANOVA). Values represent the mean % change in body weight ± S.E.M.

Handled animals had lower plasma leptin levels (F_1,45 _= 4.163 p = 0.047), (Fig. 5), consumed less food (F_1,15_= 4.580, p = 0.05), (Fig. 6), and gained less weight (F_1,15 _= 7.392, p = 0.017) during long-term forced swimming stress, compared to the non-handled (Fig. 7).

## Discussion

Neonatal handling has beneficial effects in the male rats. In addition to its well-documented effects in increasing their ability to cope with stress [2,3], our present results show that it also improves their cognitive abilities. Furthermore, handling resulted in increased hippocampal GR and decreased MR levels. The observed increase in the GR/MR ratio reflects prevalence of GR-mediated effects and implies an increased HPA axis sensitivity. It is noteworthy that the handling-induced increase in basal GR levels, shown by others, [33] persists after exposure to a short-term restraint stress, followed by the Morris water maze as shown by the present results. The superior mnemonic performance of the handled animals could be attributed to the increased levels of GR, since they are involved in the consolidation of learned information and their activation is a prerequisite for optimal memory [5].

Furthermore, our results show that handling increases BDNF. BDNF levels are known to be positively related to learning as well as to have anti-depressive effects. This is particularly interesting in relation both to the present data regarding the effects of handling on learning and memory and our previous results showing that handled males show less "depressive" behavior as assessed by shorter immobility times in the chronic forced swimming stress [26]. It thus appears that handling protects males from chronic stress-induced "depressive" behavior, possibly by increasing basal BDNF levels.

Another pathway underlying the protective effects of handling against stress could involve the serotoninergic system, since our results show that handling increases 5-HT1A receptors, which are directly involved in the action of anti-depressants. Furthermore, results from our laboratory have shown that handling also increases serotonin levels [34]. Interestingly, BDNF has been shown to have a trophic effect on serotoninergic neurons [18] and in general to interact with the serotoninergic system [17]. Among its actions presumed to be mediated through such mechanisms are the effects on appetite, body weight and plasma leptin levels [17,35]. It is noteworthy, that there is an inverse relationship between BDNF and leptin levels: BDNF conditional knockout mice exhibit hyperphagia [17] and over 15-fold higher leptin levels [35].

According to the results of the present work, during chronic forced-swimming stress non-handled males, consume more food and gain more weight compared to the handled. Furthermore, after the last exposure to the stressor, they have higher plasma leptin concentrations. These findings may be relevant to the human condition of stress-induced obesity [36,37], which is believed to be associated with glucocorticoid-induced resistance to leptin [38] accompanied by elevated leptin levels [39]. In addition to increased food intake, non-handled males showed decreased energy expenditure, as revealed by longer immobility times, during the last exposure to our chronic forced-swimming paradigm [26]. Both decreased energy expenditure and increased appetite push energy balance towards energy storage and weight gain. This could explain our results showing that during chronic-forced swimming, non-handled males gain more weight than handled males.

It has been proposed that the beneficial effects of neonatal handling are the outcome of the increased maternal care, which the handled animals receive [33]. Thus, our work provides evidence that alterations in maternal care can lead to long lasting changes in brain function affecting cognitive abilities and affective states.

## Conclusion

Handling has a beneficial effect on males, improving their cognitive abilities and reducing their propensity to express maladaptive behavior following chronic stressors. The molecular basis of these effects on behavior could involve the observed handling-induced increase in hippocampal GR/MR, BDNF, and 5-HT1A receptor levels, as well as the decrease in circulating leptinlevels.

## List of abbreviations

5-HT1A type 1A serotonin receptors

ANOVA analysis of variance

BDNF brain derived neurotrophic factor

CA1-4 fields 1–4 of Ammon's horn

DG hippocampal dentate gyrus

GR glucocorticoid receptors

HPA axis hypothalamic-pituitary-adrenal axis

MR mineralocorticoid receptors

MWM Morris watter maze

RIA radio-immuno-assay

## Competing interests

The author(s) declare that they have no competing interests.

## Authors' contributions

EG carried out the BDNF immunocytochemistry. TP carried out the body weight and food consumption measurements as well as the plasma leptin levels determination. SP carried out the spatial learning and memory tests as well as the GR and MR immunocytochemistry. AS carried out the in vitro binding for the 5HT1A receptors. EF and FS conceived, designed and coordinated the study. All authors participated in the statistical analysis of the data as well as to draft the manuscript. All authors read and approved the final manuscript.
